# Deep learning-based combined noise reduction and contrast enhancement for post-neoadjuvant pancreatic cancer CT: does improved image quality translate to better resectability assessment?

**DOI:** 10.1007/s00261-025-05271-6

**Published:** 2025-11-04

**Authors:** Seok Jin Hong, Sun Kyung Jeon, Jeongin Yoo, Liang Zhu, Jeong Hee Yoon, Hyo-Jin Kang, Junghoan Park, Jeong Min Lee

**Affiliations:** 1 Department of Radiology, Gyeongsang National University Hospital, Jinju, Korea, Republic of; 2 Department of Radiology, Seoul National University Hospital, Seoul, Korea, Republic of; 3 Department of Radiology, Peking Union Medical College Hospital, Beijing, China; 4 Department of Radiology, Seoul National University, Seoul, Korea, Republic of

**Keywords:** Deep learning, Pancreatic neoplasm, Neoadjuvant therapy, Image enhancement, Multidetector computed tomography

## Abstract

**Purpose:**

To evaluate whether deep learning-based combined noise reduction and contrast enhancement reconstruction (DLR) improves image quality and resectability prediction accuracy compared to conventional iterative reconstruction (IR) in post-neoadjuvant pancreatic cancer CT assessment.

**Methods:**

This retrospective study included 114 patients with pancreatic cancer following neoadjuvant therapy. Contrast-enhanced CT images were reconstructed using conventional IR and vendor-neutral ClariACE. Three abdominal radiologists independently assessed image quality (based on 8 parameters: tumor conspicuity, tumor margin, image noise, sharpness of the main pancreatic duct, arterial depiction, venous depiction, plasticity and overall image quality) and determined tumor resectability with confidence levels. Quantitative analysis included aortic and portal venous attenuation measurements and pancreas-to-tumor contrast-to-noise ratio (CNR). Diagnostic performance was evaluated using the area under the receiver operating characteristic curve (AUC) with DeLong’s test. Sensitivity, specificity, accuracy, and reader confidence were compared using McNemar’s test.

**Results:**

DLR demonstrated significantly superior vessel enhancement (*p* < 0.001) and improved CNR (*p* < 0.001) versus conventional IR. Two readers consistently rated DLR images higher across all qualitative categories (*p* < 0.001) except plasticity, while the third reader favored DLR in five of eight parameters (*p* < 0.001 to 0.020). However, all readers noted increased artificial appearance in DLR images (*p* < 0.001). Despite image quality improvements, no significant differences were observed in resectability assessment accuracy (62.3%-65.8%), AUC values (0.485–0.520), or high-confidence diagnosis rates between reconstruction methods.

**Conclusion:**

Although deep learning-based combined noise reduction and contrast enhancement reconstruction significantly improved quantitative and subjective image quality metrics, it did not enhance diagnostic accuracy for predicting R0 resectability in post-neoadjuvant pancreatic cancer patients.

## Introduction

Neoadjuvant therapy has become the standard of care for borderline resectable and locally advanced pancreatic ductal adenocarcinoma (PDAC), with the potential to convert unresectable tumors to resectable status and improve long-term survival outcomes [1–3] ​. Accurate imaging assessment following neoadjuvant therapy guides treatment decisions by determining which patients can achieve an R0 resection (complete tumor removal with negative margins) [1, 4]. However, accurate assessment of treatment response and surgical resectability after neoadjuvant therapy represents one of the most challenging problems in contemporary pancreatic imaging [5, 6]. The fundamental difficulty stems from the biological effects of chemotherapy and radiation therapy on pancreatic tissue. Treatment induces extensive desmoplastic reaction and fibrosis through activation of pancreatic stellate cells, creating tissue changes that are morphologically indistinguishable from tumor-associated desmoplasia on conventional imaging [7, 8]. This treatment-induced fibrosis can persist for months after therapy completion, leading to systematic overestimation of residual tumor burden and potential denial of curative surgery to appropriate candidates [5, 9, 10].

Accurate imaging assessment plays a pivotal role in resectability determination; however, the radiological differentiation between persistent viable tumor and post-treatment fibrotic tissue, coupled with the prediction of microscopic margin involvement, represents a persistent diagnostic challenge. Furthermore, the identification of subtle hypovascular pancreatic lesions and the precise characterization of tumor-vessel interface relationships remain technically demanding in post-neoadjuvant imaging evaluation [11]. Multiple studies have demonstrated that conventional CT accuracy for predicting successful R0 resection drops dramatically after neoadjuvant therapy. While CT achieves 83% accuracy in treatment-naive pancreatic cancer, accuracy falls to just 58% in the post-neoadjuvant setting [11, 12]. This accuracy degradation directly impacts clinical decision-making, as radiological assessment often drives multidisciplinary team recommendations regarding surgical candidacy [13].

Conventional contrast-enhanced pancreatic CT employs iterative reconstruction (IR) techniques to reduce noise and improve image quality [14]. New approaches to enhance tumor and vessel visualization include dual-energy CT, which can generate low-keV virtual monochromatic images that accentuate iodine contrast, improving tumor-to-pancreas contrast and vessel clarity [15, 16]. However, dual-energy CT requires specialized scanners, limiting its widespread application. Deep learning algorithms have recently emerged as a promising alternative to boost iodine contrast in CT images post-acquisition. ClariACE (ClariCT.ACE, ClariPi) is a vendor-neutral deep learning-based noise reduction and contrast-boosting reconstruction (DLR) algorithm [17]. Prior studies in liver imaging have demonstrated that such DLR technique can significantly improve image contrast and quality, even when using reduced contrast media doses [18–20]. Given the well-documented limitations of conventional CT in the post-neoadjuvant setting, we hypothesized that advanced AI-based reconstruction techniques combining both noise reduction and contrast enhancement might improve diagnostic performance where conventional techniques fail. Unlike previous studies that evaluated AI reconstruction in treatment-naive pancreatic cancer [21, 22], our study specifically focuses on the post-chemotherapy setting where imaging challenges are most pronounced and clinical need is greatest.

Thus, our study evaluated whether DLR improves image quality and – critically – whether it improves the accuracy and confidence of resectability predictions compared to conventional CT using IR.

## Materials and methods

This retrospective study was approved by the Institutional Review Board of out institution (IRB No. 2408-035-1558), which waived the requirement for obtaining informed consent. Financial support was provided by ClariPi; however, the authors had complete control of the data and information submitted for publication at all times.

### Patients

**Fig. 1:**
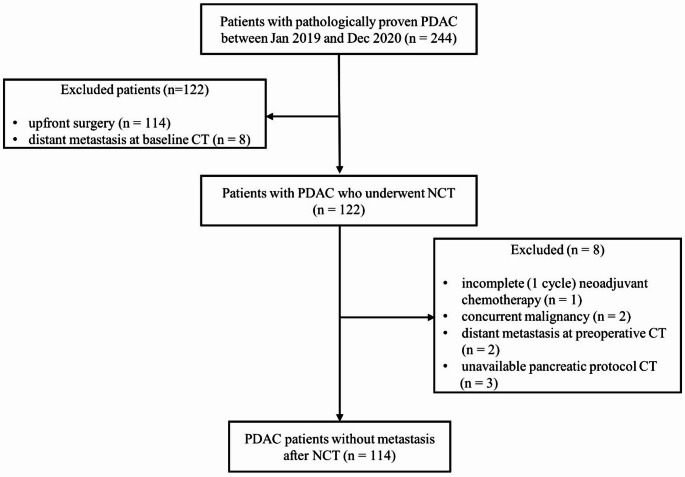
Flow chart of the study population. PDAC: pancreatic ductal adenocarcinoma, NCT: neoadjuvant chemotherapy

Patients who met the eligibility criteria between January 2019 and December 2020 were included: (1) pathologically confirmed PDAC, (2) completion of neoadjuvant therapy (FOLFIRINOX or gemcitabine-based regimen, minimum 4 cycles), (3) surgical resection within 8 weeks of CT examination. Exclusion criteria were as follows: (1) presence of distant metastases, (2) other concurrent malignancies, (3) poor CT image quality (motion artifacts, incomplete contrast enhancement), (4) incomplete neoadjuvant therapy course (Fig. 1).

## CT technique

All CT scans were acquired with a multiphasic contrast-enhanced technique that included unenhanced, early arterial, pancreatic, and venous phases. Precontrast images were acquired first. The early arterial phase was initiated using a bolus-tracking technique, with image acquisition triggered automatically 6–9 s after the attenuation of the descending aorta reached 100 Hounsfield unit (HU). The average delay time for the early arterial phase acquisition was 23 s after triggering. Subsequently, the pancreatic and venous phases were obtained at 40 and 70 s after triggering, respectively.

CT scans were performed with the following parameters: tube voltage of 120 kVp, tube current of 150–250 mAs, slice thickness of 2–3 mm, reconstruction interval of 0.6–5 mm, pitch of 0.9-1.0, and gantry rotation time of 0.5–1.0 s. Contrast-enhanced scans were obtained following intravenous injection of iobitridol (Xenetix 350, Guerbet) or isohexol (Bonorex 350, Central Medical Service) at a dose of 1.5 mL/kg body weight using an automatic power injector (Stellant Dual, Medrad) at a rate of 2.0–5.0 mL/s, followed by a 20-mL saline flush.

Multidetector computed tomography (MDCT) examinations were performed using the following scanners: 320-MDCT (Aquilion ONE, Canon Medical Systems) (*n* = 2), 256-MDCT (Brilliance iCT, Philips Healthcare; Revolution, GE Healthcare) (*n* = 24), 192-MDCT (Somatom Force, Siemens Healthineers) (*n* = 36), 128-MDCT (Ingenuity, Philips Healthcare; IQon Spectral, Philips Healthcare) (*n* = 33), and 64-MDCT (Brilliance 64, Philips Healthcare; Somatom Definition, Siemens Healthineers; or Discovery CT750 HD, GE Healthcare) (*n* = 19).

After CT image acquisition, the DLR algorithm was applied using ClariACE (ClariCT.ACE, ClariPi, Seoul, Korea), which employs a two-stage U-Net convolutional neural network that first denoises and then amplifies iodine signals in standard CT images [18–20, 23]. The algorithm had been previously trained on more than one million pairs of dual-energy CT images from multiple vendors, including various contrast phases and dose levels, thereby incorporating datasets with imaging characteristics similar to those in our study. By extracting and enhancing the iodine distribution from the original images, the algorithm generates contrast-boosted CT images that simulate the visual effect of lower-keV acquisition with higher iodine attenuation [18]. The output images are expressed in Hounsfield unit (HU), consistent with standard CT, and should be regarded as enhanced standard images rather than reconstructed virtual monoenergetic images.

## Image analysis

### Quantitative analysis

**Fig. 2:**
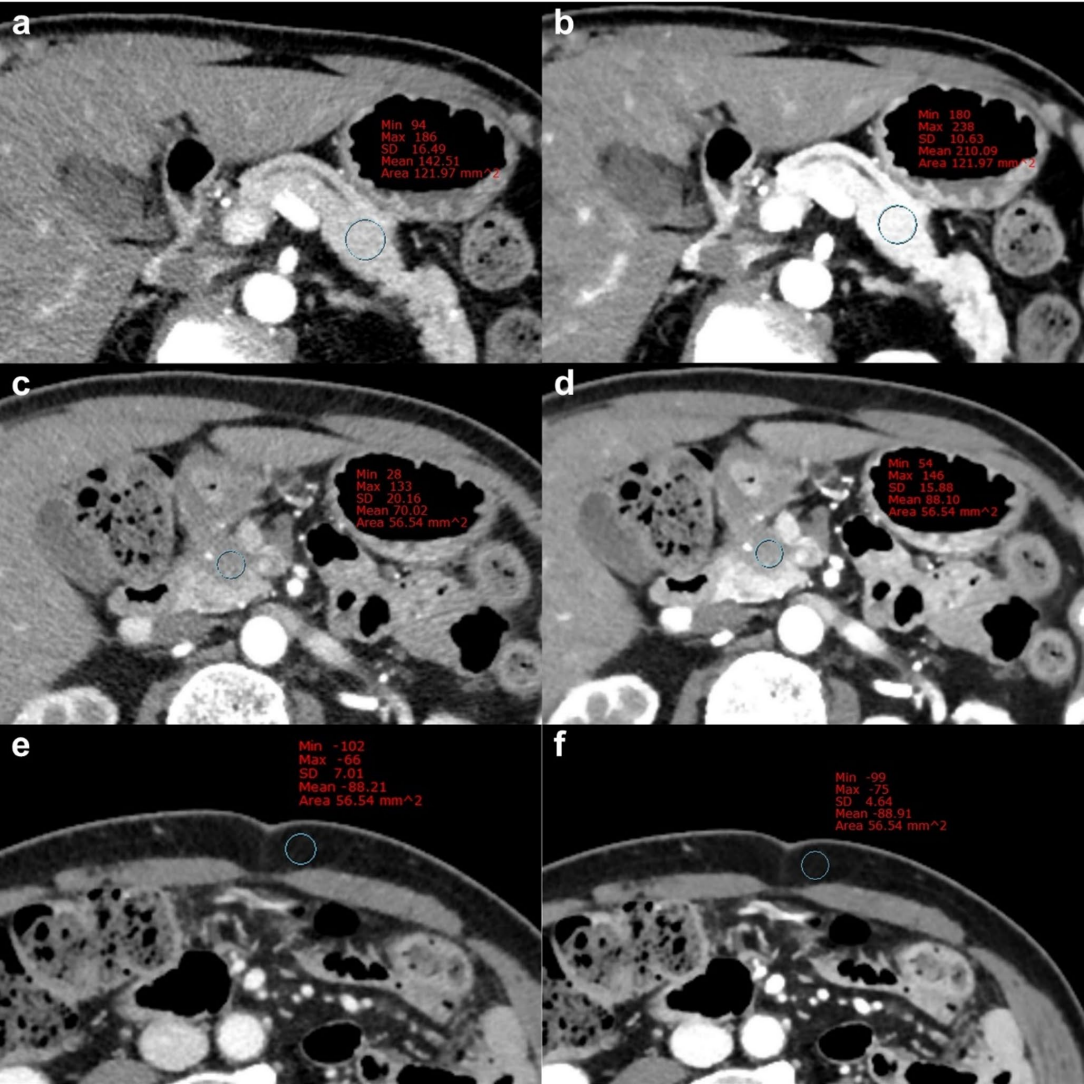
Contrast-to-noise ratio (CNR) measurement methodology demonstrated in a 64-year-old male patient with pancreatic ductal adenocarcinoma using conventional iterative reconstruction and DLR. Region of interest placement for pancreatic parenchyma attenuation measurement on pancreatic phase CT in conventional iterative reconstruction image (**a**, 142.51 HU) and DLR image (**b**, 210.09 HU). Region of interest placement for pancreatic adenocarcinoma attenuation measurement on pancreatic phase CT in conventional iterative reconstruction image (**c**, 70.02 HU) and DLR image (**d**, 88.10 HU). Image noise assessment via SD measurement of subcutaneous fat attenuation on portal phase CT in conventional iterative reconstruction image (**e**, 7.01 SD) and DLR image (**f**, 4.64 SD). CNR calculation: (pancreatic parenchyma attenuation - tumor attenuation)/image noise. In conventional iterative reconstruction image: CNR = (142.51–70.02)/7.01 = 10.34. In DLR image: CNR = (210.09–88.10)/4.64 = 26.29. DLR: deep learning-based noise reduction and contrast-boosting reconstruction, HU: Hounsfield unit, SD: standard deviation

One author (S.J.H., 6 years of radiology experience) performed quantitative measurements on both the conventional and DLR images (Fig. 2). The Hounsfield unit (HU) of the abdominal aorta, pancreatic parenchyma and tumor were evaluated in pancreatic phase image. Measurements were conducted by manually outlining regions of interest (ROI) on the axial CT scan. Careful ROI selection was performed to avoid areas containing cystic portions, necrosis, vessels, dilated ducts, or fat-containing regions. The HU of the main portal vein (MPV) was obtained in the venous phase. Noise was defined as the mean standard deviation of subcutaneous fat attenuation measured at the level of the umbilicus in the anterior abdominal wall. The parenchyma-to-tumor contrast-to-noise ratio (CNR) was calculated according to the following equation.

CNR = (Mean HU of pancreas parenchyma – Mean HU of pancreatic tumor) / noise.

All measurements were performed three times and then averaged.

## Qualitative analysis

Qualitative assessments were performed on both conventional and DLR images by three board-certified abdominal radiologists: two reviewers with 5 years of experience each, and one reviewer with 8 years of experience. These assessments were conducted with blinding to the image reconstruction type, and the clinical outcome, and the images were randomly distributed. Seven parameters (tumor conspicuity, tumor margin, image noise, sharpness of the main pancreatic duct, arterial depiction, venous depiction, overall image quality) were rated on a 5-point scale, and image plasticity was assessed on a 4-point scale. The specific scoring criteria were as follows:

Tumor conspicuity: 1 = No distinct tumor, 2 = Poorly distinct tumor, 3 = Average, 4 = Good, 5 = Excellent.

Tumor margin: 1 = Unclear, 2 = Poorly demarcated tumor, 3 = Average, 4 = Good, 5 = Excellent.

Vessel depiction and sharpness of the MPD: 1 = Blurry, 2 = Suboptimal, 3 = Average, 4 = Above average, 5 = Excellent.

Image noise: 1 = Undiagnostic, 2 = Suboptimal, 3 = Moderate, 4 = Mild, 5 = Absent.

Plasticity: 1 = Extremely plastic, 2 = Moderately plastic, 3 = Mildly plastic, 4 = Non-plastic.

### Assessment of PDAC resectability

Three board-certified abdominal radiologists—Y.J.I and J.S.K and J.L —evaluated both conventional and DLR images. To ascertain PDAC resectability for each participant, data using axial and coronal CT images concerning the tumor’s location, size, variation of common hepatic artery and tumor vascular contact were collected and recorded. The reviews for resectability assessment were conducted independently by all three reviewers, with blinding to patients’ clinical information and image reconstruction type. The images were randomly distributed. The tumor-vessel relationship was evaluated for the celiac trunk, common hepatic artery, and superior mesenteric artery; and for veins, the superior mesenteric vein, main portal vein, and inferior vena cava were assessed. Only direct contact between the tumor and vessel was considered valid, and hazy attenuation or non-enhancing infiltration around the vessel was not regarded as tumor contact [23, 24]. The tumor-vessel relationship was categorized into no contact, abutment (< 180°), encasement ( ≧ 180°), vascular deformity, and tumor thrombus. If there was encasement, vascular deformity, or tumor thrombus in the vessel, it was assessed as vessel invasion. For the SMV and MPV, if tumor invasion led to occlusion or if there was an invasion of 3 cm or more, it was classified as non-reconstructable [25]. Based on these imaging findings, each case was assigned an NCCN (Pancreatic Adenocarcinoma, Version 3.2024) resectability category (resectable, borderline resectable, or locally advanced) [26, 27]. A corresponding 5-point confidence score was also recorded: 1, definitely unresectable; 2, probably unresectable; 3, borderline resectable; 4, probably resectable; and 5, definitely resectable.

## Determination of PDAC resectability: reference standard

PDAC resectability was primarily determined by surgical and histopathologic findings, which served as the reference standard. R0 indicated no residual tumor on microscopic assessment; R1 indicated no gross residual tumor, but tumor cells present within 1 mm of the resection margin or at the cut surface; and R2 indicated the presence of gross residual tumor.

### Statistical analysis

Continuous variables were presented as medians with interquartile ranges (IQRs). Ordinal variables from qualitative assessments were summarized using means and standard deviations for descriptive purpose. To compare qualitative assessments between conventional and DLR images, the Wilcoxon signed-rank test was performed. To evaluate the diagnostic performance of conventional and DLR images in predicting R0 resection for PDAC, the area under the receiver operating characteristic curve (AUC) was calculated and compared using DeLong’s test. Sensitivity, specificity, and accuracy for predicting R0 resection were also computed for both protocols and compared using McNemar’s test. For statistical analysis of reader confidence, responses were dichotomized into a high-confidence group (definite resectable or unresectable) and a low-confidence group (probable resectable or unresectable and borderline resectable), and comparisons between conventional and DLR images were performed using McNemar’s test. Inter-reader agreement in assessing R0 resection probability was assessed using kappa statistics and categorized as poor (< 0.20), fair (0.20–0.40), moderate (0.40–0.60), good (0.60–0.80), or excellent (0.80–1.00). All analyses were performed using R software (version 4.4.3; http://www.R-project.org).

## Results

### Patients’ characteristics

Of 114 patients included in the study, 59 (51.8%) were male and 55 (48.2%) were female, with a median age of 64 years (IQR, 49.0–71.0 years). Based on pathological records, R0 resection was achieved in 78 patients (68.4%), R1 in 25 patients (21.9%), and R2 in 11 patients (9.6%).

The location of the pancreatic cancer was the head and/or uncinate process in 70 cases (61.4%), the body in 33 cases (29%), and the tail in 11 cases (9.6%). The median size of the pancreatic cancer was 2 cm (IQR, 1.6–2.4 cm). Patients’ characteristics are summarized in Table 1.

**Table 1:** Demographic and clinical characteristics of study participants

Characteristics	Total (*n* = 114)
Age (IQR^a^, years)	64.0 [59.0;71.0]
Sex (male: female)	59 (51.8%):55 (48.2%)
Resection margin status	
R0	78 (68.4%)
R1 or R2	36 (31.6%)
Tumor location	
head/uncinate process	70 (61.4%)
body	33 (28.9%)
tail	11 (9.6%)
Size (IQR, cm)	2.0 [1.6;2.4]

IQR^a^: interquartile range

### Quantitative assessment

**Fig. 3:**
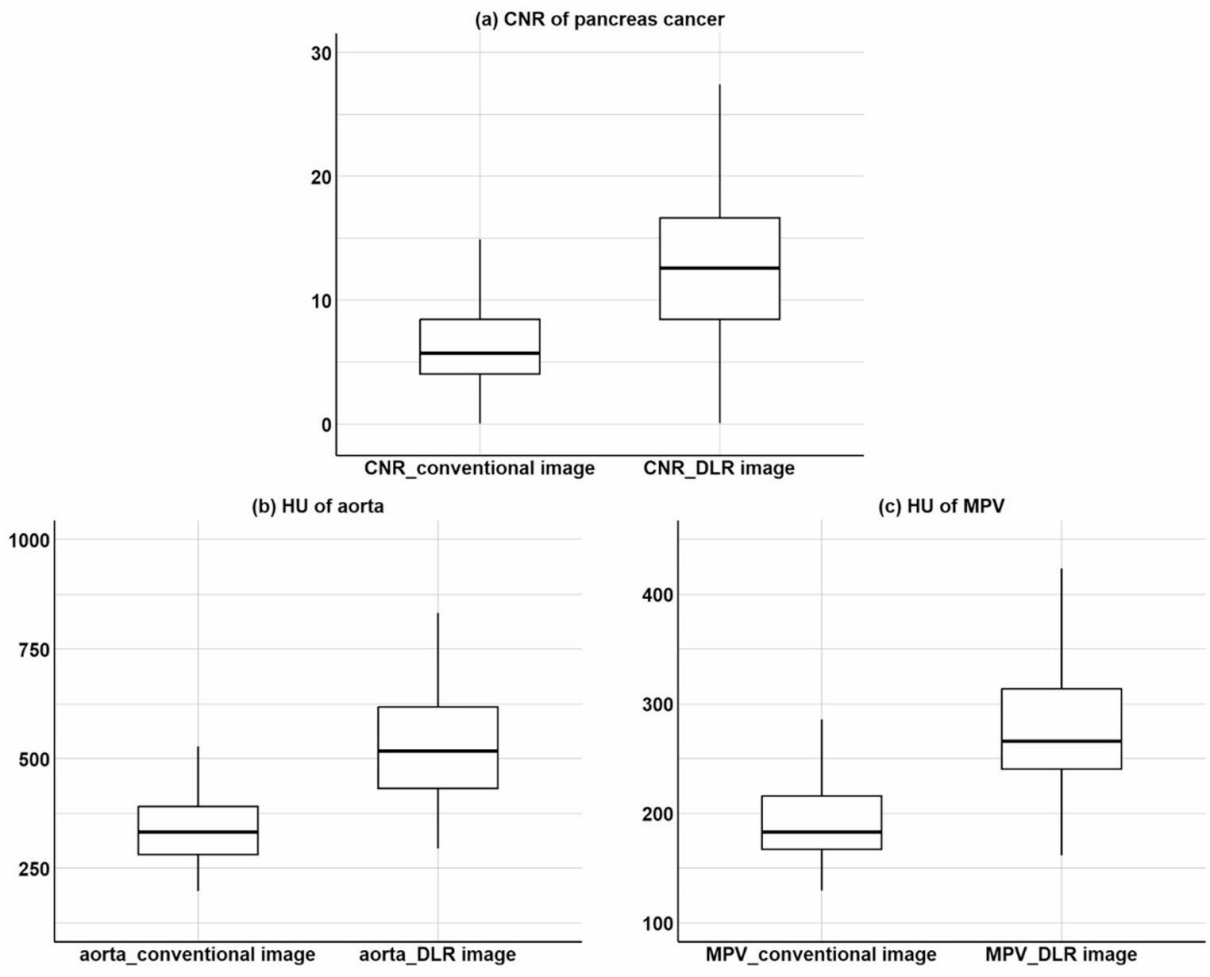
Quantitative image quality comparison between conventional iterative reconstruction and DLR. Box plots demonstrate (**a**) pancreatic cancer contrast-to-noise ratio (CNR), (**b**) abdominal aortic attenuation in arterial phase (Hounsfield unit), and (**c**) main portal vein attenuation (Hounsfield unit) All quantitative metrics demonstrated statistically significant improvement with DLR (*p* < 0.001). DLR: deep learning-based noise reduction and contrast-boosting reconstruction

Median pancreas-to-tumor CNR increased from 5.7 (IQR 4.1–8.4) with conventional image to 12.6 (IQR 8.5–16.6) with DLR image (*p* < 0.001) (Fig. 3a). Similarly, the median attenuation of the abdominal aorta in the arterial phase rose from 332 HU (IQR 281–390 HU) with conventional image to 517 HU (432–617 HU) with DLR image (*p* < 0.001) (Fig. 3b). The median attenuation of the MPV in the portal phase was 183 HU (IQR, 167–216 HU) with conventional image and 266 HU (IQR, 241–314 HU) with DLR image (*p* < 0.001) (Fig. 3c).

### Qualitative assessment

**Fig. 4:**
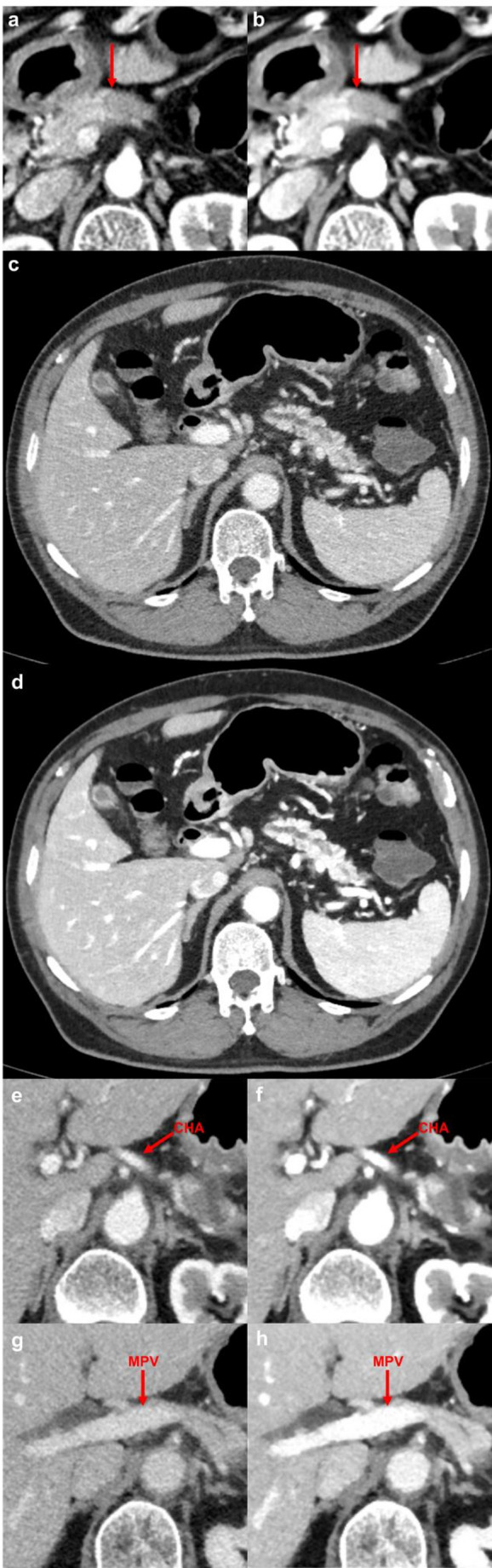
Representative examples demonstrating qualitative image parameter improvements with DLR compared to conventional iterative reconstruction. (**a**,** b**) Tumor margin (arrow) clarity assessment in a 68-year-old female patient: conventional reconstruction (score 3, average) versus DLR (score 5, excellent). (**c**,** d**) Image noise evaluation in a 70-year-old male patient: conventional reconstruction (score 2, suboptimal) versus DLR (score 4, mild noise). (**e-h**) Vascular depiction assessment in a 65-year-old female patient demonstrating CHA (common hepatic artery, arrows in **e**,** f**) and MPV (main portal vein, arrows in **g**,** h**): conventional reconstruction (**e**,** g**; score 2, suboptimal) versus DLR (**f**,** h**; score 4, above average). DLR: deep learning-based noise reduction and contrast-boosting reconstruction

DLR significantly improved most qualitative image scores. Two of three readers rated DLR images higher in all categories (*p* < 0.001) except plasticity, and the third reader also favored DLR images for tumor conspicuity (*p* = 0.007), artery depiction (*p* = 0.02), vein depiction (*p* = 0.014), and lower noise (*p* < 0.001). Mean improvements for all comparisons ranged from 0.2 to 1.2 points on a 5-point scale. Notably, in the ‘plasticity’ category (image texture), all readers found DLR images more artificial-looking (more “plastic”) than conventional CT (*p* < 0.001). Figure 4 illustrates qualitative differences in tumor margin, noise and vessel depiction between conventional and DLR images. Representative examples of the remaining four assessment categories are provided in Appendix S1.

### Assessment of PDAC resectability

Despite enhanced image quality, DLR reconstruction did not improve diagnostic performance for predicting R0 resection. All three readers’ sensitivities, specificities, and overall accuracies for R0 vs. R1/R2 were statistically similar between DLR and conventional images. Reader confidence in resectability calls was unchanged (reader1, *p* = 0.61; reader2, *p* = 1; reader3, *p* = 0.81, Table 3). The AUC, sensitivity, specificity, and accuracy of the diagnostic performance for each reader are summarized in the Table 2.

**Table 2:** Comparison of diagnostic performance metrics between conventional iterative reconstruction and *DLR* images for R0 resection prediction by three readers

		Conventional images	DLR images	*p*-value
Reader 1	AUC^a^	0.520	0.515	0.899
	Sensitivity	36.8% (7/19)	34.8% (8/23)	1
	Specificity	69.5% (66/95)	69.2% (63/91)	0.505
	Accuracy	64.0% (73/114)	62.3% (71/114)	0.453
Reader 2	AUC	0.503	0.503	1
	Sensitivity	33.3% (3/9)	33.3% (3/9)	1
	Specificity	68.6% (72/105)	68.6% (72/105)	1
	Accuracy	65.8% (75/114)	65.8% (75/114)	1
Reader 3	AUC	0.485	0.504	0.462
	Sensitivity	26.7% (4/15)	33.3% (4/12)	1
	Specificity	67.7% (67/99)	68.6% (70/102)	0.449
	Accuracy	62.3% (71/114)	64.9% (74/114)	0.505

AUC^a^: area under the receiver operating characteristic curve

### Inter-reader agreement

Inter-reader agreement on resectability was moderate to good with conventional images (κ = 0.44–0.63 across reader pairs) and fair to moderate with DLR images (κ = 0.30–0.53). All 95% confidence intervals for κ overlapped, indicating no significant difference in agreement levels between the techniques.

## Discussion

In this study, we demonstrated that DLR significantly improved the image quality of pancreatic CT scans compared to conventional IR. Specifically, DLR provided superior enhancement of pancreatic tumors, major vessels, and reduced image noise, yielding a 120% increase in tumor-to-parenchyma CNR from 5.7 to 12.6 (Fig. 3a). Consistently higher qualitative scores for tumor conspicuity, vessel depiction, and overall image clarity further underscore these advantages (Fig. 4). These findings align with previous findings in liver imaging, where deep-learning reconstructions enhanced image contrast and CNR even under conditions of reduced iodine dose [15, 18, 20, 21].

Despite these clear improvements in image quality, our results indicate that enhanced visualization did not translate into improved diagnostic performance or confidence in assessing tumor resectability (Tables 2 and 3). Both conventional and DLR images exhibited similar accuracy (approximately 64–66%) and modest performance metrics (AUC ranging from 0.485 to 0.520) for predicting R0 resection status, as demonstrated in Table 2. This indicates that while image quality is crucial for radiological interpretation, high image quality alone is insufficient for accurate prediction of surgical margins in pancreatic cancer patients who have undergone neoadjuvant therapy.

**Fig. 5:**
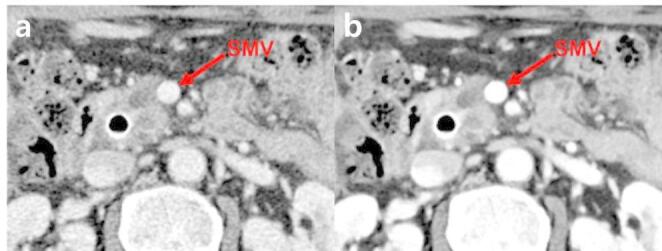
A 54-year-old male patient with pancreatic ductal adenocarcinoma (PDAC) who ultimately underwent R2 resection at interface of the superior mesenteric vein (SMV) following neoadjuvant therapy. (**a**) Conventional image shows ambiguous tumor margin around the SMV (arrow) (**b**) DLR image demonstrates improved tumor conspicuity and margin definition with reduced noise (arrow). Despite superior image quality, inter-reader resectability assessments remained discordant and unchanged across both reconstruction methods: Reader 1 classified the tumor as borderline resectable on both images, while Readers 2 and 3 classified it as resectable, highlighting the limitations of image quality enhancement for accurate resectability prediction. DLR: deep learning-based noise reduction and contrast-boosting reconstruction

Several biological and clinical factors may explain this apparent disconnect between improved image quality and diagnostic performance. First, the definition of “resectability” in pancreatic cancer after neoadjuvant treatment is inherently complex and not solely determined by imaging clarity. Borderline resectable tumors by definition have equivocal vessel involvement—even with perfect image contrast, small amounts of tumor infiltration versus inflammatory fibrosis at the margin can appear similar on CT [23]. The ultimate determination of an R0 resection often depends on microscopic tumor invasion, which no current imaging modality can reliably detect (Fig. 5). Many patients deemed “resectable” on imaging still had microscopically positive or close margins (R1/R2) at surgery, indicating that the limiting factor was not the gross visibility of the tumor-vessel interface but rather occult tumor cells beyond CT resolution capabilities. Conversely, some tumors classified as “borderline” on CT were successfully resected with clean margins, suggesting that imaging signs of vessel abutment did not preclude safe resection. These scenarios depend more on tumor biology and treatment response than on absolute CT contrast enhancement [9, 11]. This likely contributed to the moderate inter-reader agreement observed (κ ranging from 0.3 to 0.6) and the lack of improvement with enhanced images.

Second, readers have assessed resectability primarily based on the tumor–vessel relationship. However, factors contributing to R0 resection include not only the tumor-vessel interface but also tumor size, tumor location, invasion of adjacent organs, and the type of surgical procedure employed [4, 22]. In fact, within our study group, among the 31.6% (36/114) of patients who failed to achieve R0 resection, only 33.3% (12/36) had tumor involvement at the tumor-vessel interface, whereas 66.7% (24/36) had close (< 1 mm) or involved margins at the anterior or posterior surfaces of the tumor, unrelated to vessel involvement. These findings underscore the limitation of assessing pancreatic cancer resectability using only the tumor-vessel interface. Given these methodological limitations, accurately predicting PDAC resectability based solely on radiologic findings is challenging; therefore, the NCCN guidelines recommend that the final decision on resectability status be reached by consensus in a multidisciplinary discussion [26].

Our findings regarding image quality improvement with the DLR algorithm are consistent with recent research evaluating DLR in other clinical contexts. Kang et al. [19], examining low-contrast-dose liver CT, reported that while a DLR model improved vessel CNR and image quality, it did not significantly change lesion detection rates. Similarly, Yoon et al. [21] found no difference in lesion conspicuity between standard and deep learning-augmented images of low-iodine liver CT, despite higher image contrast with the deep learning technique. These studies suggest that once a certain baseline image quality is achieved, further enhancements may yield diminishing returns for diagnostic tasks—especially when those tasks are limited by factors other than image clarity.

It is worth noting that one prior study did find a diagnostic gain with DLR: Lim et al. [20] reported improved detection of small hepatic lesions when using a similar contrast-boosted model in a low-dose CT setting. In that case, the algorithm compensated for suboptimal contrast dose, actually yielding better lesion visibility than a standard full-contrast scan. By comparison, in our study the standard scan already had adequate contrast for imaging the pancreas, so DLR was enhancing an already diagnostic-quality image. In addition, evaluating resectability considering extent of the tumor is more complex than lesion detection, as previously mentioned. This may explain why we observed no further improvement in characterizing tumor extent—the added contrast was somewhat redundant for decision-making, even if it enhanced visual appeal.

**Fig. 6:**
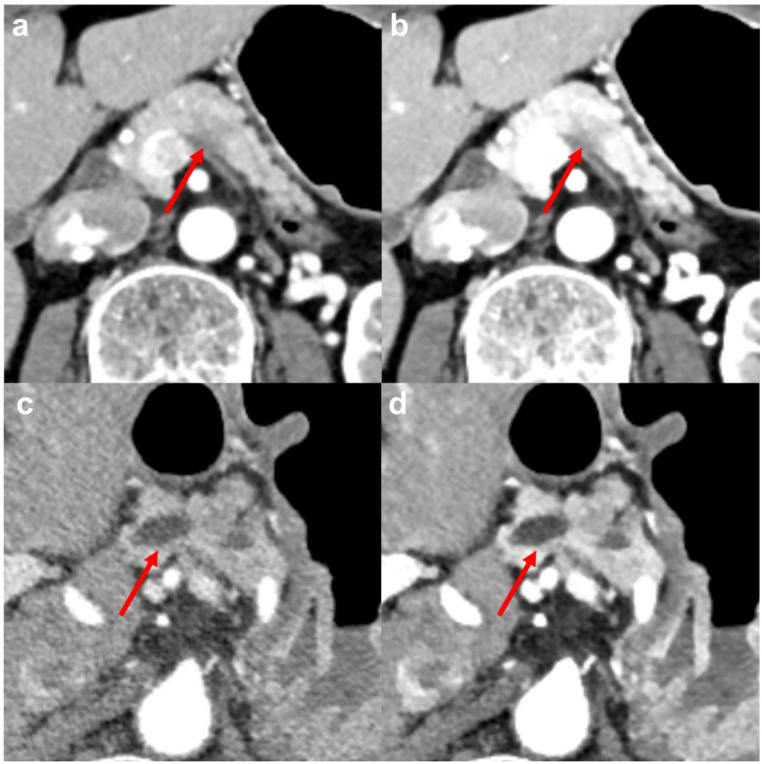

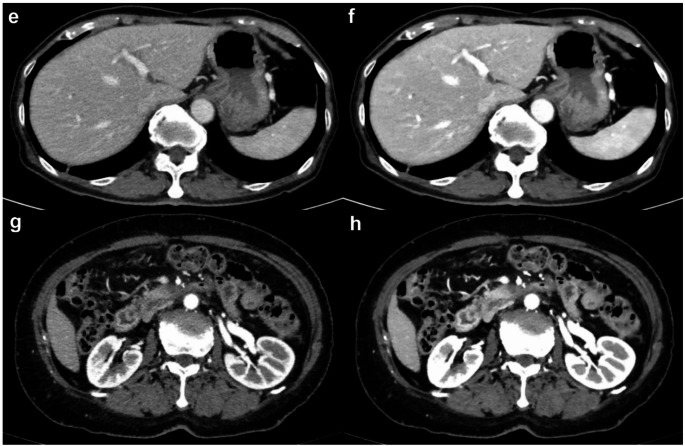
Representative examples of qualitative comparison between conventional iterative reconstruction and DLR images. (**a**,** b**) Tumor (arrow) conspicuity in a 56-year-old male patient: conventional reconstruction (score 2, poorly distinct tumor) versus DLR (score 4, good). (**c**,** d**) Sharpness of the main pancreatic duct (arrow) in a 64-year-old male patient: conventional reconstruction (score 3, average) versus DLR (score 5, sharpest). (**e**,** f**) Plasticity in a 78-year-old female patient: conventional reconstruction (score 4, non-plastic) versus DLR (score 4, good). (**g**,** h**) Overall image quality in a 72-year-old female patient: conventional reconstruction (score 3, average) versus DLR (score 5, excellent). DLR: deep learning-based noise reduction and contrast-boosting reconstruction

One notable trade-off observed with the DLR was the artificially smooth or “plastic” image texture reported by all readers (Fig. 6e-f; Table 4). While DLR improved objective and subjective image quality scores, radiologists noted a more artificial texture in these images. This “plastic” appearance is a known phenomenon with aggressive noise reduction algorithms, including AI-based reconstructions [19, 21]. In our study, readers rated conventional images as less artificial-looking than DLR images, suggesting that DLR may oversmooth or alter some image details while boosting contrast and removing noise (Table 4). It is possible that this artificial texture could obscure very fine details or subtle cues that radiologists subconsciously use in assessment, potentially counteracting some benefits of enhanced contrast. Achieving optimal balance between noise reduction, contrast enhancement, and preservation of natural image texture remains an ongoing challenge for developers of deep-learning reconstruction algorithms.

In a previous study evaluating the diagnostic performance of pancreatic resectability using DLR, high-strength DLR images demonstrated superior diagnostic performance compared with filtered back projection and IR images [28]. In that study, high-strength DLR improved overall image quality by reducing image noise, enhancing noise texture and low-contrast spatial resolution, and improving sharpness and lesion conspicuity. These advantages translated into improved diagnostic performance for assessing the resectability of PDAC. While Lyu et al. reported improved diagnostic performance with DLR, critical methodological differences explain the discrepant findings: their cohort included initially resectable cases and unconfirmed unresectable cases (11/15 without surgery), whereas our study exclusively examined the most challenging scenario—post-neoadjuvant PDAC with surgical confirmation in all cases.

From a clinical standpoint, while DLR images may not increase the likelihood of correctly predicting an R0 resection, it could still provide value by delivering higher-quality images without additional change of CT protocol, contrast or radiation [18–20]. Its ability to boost contrast might permit reduced contrast doses in vulnerable patients (e.g., those with renal impairment), an important consideration in pancreatic cancer care where patients often undergo multiple imaging studies [18–20]. Some benefits of improved imaging might not have been captured by our study endpoints. All readers subjectively preferred the DLR images for their clarity (Table 4), which could potentially reduce fatigue or increase satisfaction during clinical work, even without measurable accuracy improvements. There may also be clinical scenarios where better contrast could make a meaningful difference—for instance, in detecting small metastatic liver lesions or lymph nodes, though our study focused solely on local resectability assessment.

Several important limitations must be acknowledged that affect the interpretation and generalizability of our findings. First, as a retrospective analysis at a single tertiary center, the selected cohort of PDAC patients that proceeded to surgery after neoadjuvant therapy may limit generalizability. The relatively low R0 resection rate (68.4%) suggests selection of particularly challenging cases. Second, our reference standard (R0 vs. R1/R2 surgical outcome) is clinically relevant but imperfect, as margin status can be influenced by surgical technique and intraoperative decision-making, not solely by imaging-invisible tumor. Third, the moderate experience level of the radiologists (6 years on average) is also mentioned, with the suggestion that more specialized expertise might yield different results, although the moderate inter-observer agreement even with standard CT points to the inherent case difficulty. In addition, the readers had low experience with DLR images (two years for two reviewers and no prior experience for one reviewer), which may also have influenced the results. Fourth, this study evaluated only post-treatment CT scans without comparison to baseline pre-treatment imaging without integration of clinical factors such as CA 19 − 9 trends, which prevents assessment of treatment response or longitudinal changes. Fifth, we evaluated only one deep learning reconstruction algorithm (ClariACE); other tools might perform differently in balancing image enhancement and diagnostic yield. Finally, our study is the relatively heterogeneous cohort, which may reduce methodological rigor and complicate interpretation. However, this heterogeneity also reflects real-world clinical practice, where patient conditions are diverse, thereby enhancing the clinical relevance of our findings.

In conclusion, DLR significantly improved image quality in pancreatic cancer CT scans over standard IR, but this did not result in an improvement in resectability prediction performance. High-contrast resolution imaging alone appears insufficient to overcome the intrinsic challenges of post-therapy pancreatic cancer evaluation, where microscopic tumor spread and treatment-induced changes limit the accuracy of even high-quality CT imaging.

## Data Availability

The data supporting the findings of this study are available from the corresponding author upon reasonable request. To protect participant confidentiality, the data will be shared in a de-identified format.

## Appendix

See Fig. 6 and Tables 3, 4.

**Table 3:** Reader confidence in predicting pancreas ductal adenocarcinoma resectability

		Low confidence^a^	High confidence^b^	*p*-value
Reader1	Conventional IR^c^ image	87/114 (76.3%)	27/114 (23.7%)	0.6056
	DLR^*d*^ image	90/114 (78.9%)	24/114 (21.1%)	
Reader2	Conventional IR image	73/114 (64.0%)	41/114 (36.0%)	1
	DLR image	73/114 (64.0%)	41/114 (36.0%)	
reader3	Conventional IR image	73/114 (64.0%)	41/114 (36.0%)	0.8137
	DLR image	75/114 (65.8%)	39/114 (34.2%)	

Low confidence^a^: probable resectable, probable unresectable and borderline resectable

High confidence^b^: definite resectability and definite unresectable

IR^c^: iterative reconstruction

DLR^d^ deep learning-based noise reduction and contrast-boosting reconstruction

**Table 4:** Qualitative assessment scores by three readers

		Conventional image	DLR^a^ image	*p*-value
Reader1	Tumor conspicuity	3.3 ± 1	3.5 ± 0.9	0.007
	Tumor margin	2.9 ± 0.7	3 ± 0.7	0.417
	Noise	3.5 ± 0.6	4.2 ± 0.8	< 0.001
	Sharpness of MPD^b^	3.6 ± 0.8	3.7 ± 0.9	0.118
	Depiction of artery	4.9 ± 0.2	5 ± 0.1	0.020
	Depiction of vein	4.9 ± 0.3	5 ± 0.2	0.014
	Plasticity	4 ± 0.1	3.5 ± 0.6	< 0.001
	Overall quality	3.8 ± 0.6	4.3 ± 0.7	< 0.001
Reader2	Tumor conspicuity	2.8 ± 0.9	3.6 ± 1.1	< 0.001
	Tumor margin	2.6 ± 0.8	3.4 ± 1.2	< 0.001
	Noise	3.2 ± 0.8	4.4 ± 0.6	< 0.001
	Sharpness of MPD	3.1 ± 0.5	3.6 ± 0.8	< 0.001
	Depiction of artery	3.4 ± 0.6	4.5 ± 0.6	< 0.001
	Depiction of vein	3.4 ± 0.6	4.5 ± 0.6	< 0.001
	Plasticity	3.9 ± 0.4	3.2 ± 0.7	< 0.001
	Overall quality	2.9 ± 0.5	4 ± 0.7	< 0.001
Reader3	Tumor conspicuity	3.1 ± 1	3.8 ± 1.1	< 0.001
	Tumor margin	2.8 ± 1	3.5 ± 1.1	< 0.001
	Noise	3.4 ± 0.8	4.2 ± 0.8	< 0.001
	Sharpness of MPD	3.6 ± 0.8	4 ± 1	< 0.001
	Depiction of artery	4.2 ± 0.8	4.9 ± 0.3	< 0.001
	Depiction of vein	3.8 ± 0.9	4.8 ± 0.5	< 0.001
	Plasticity	3.4 ± 0.8	3.1 ± 0.8	< 0.001
	Overall quality	3.6 ± 0.9	4.4 ± 0.7	< 0.001

Values are presented as mean ± standard deviation

DLR^a^: deep learning-based noise reduction and contrast-boosting reconstruction

MPD^b^: main pancreatic duct
